# Authors’ Response to Peer Reviews of “Exploring the Reasons for Low Cataract Surgery Uptake Among Patients Detected in a Community Outreach Program in Cameroon: Focused Ethnographic Mixed Methods Study”

**DOI:** 10.2196/38899

**Published:** 2022-06-09

**Authors:** Mathew Mbwogge, Henry Ebong Nkumbe

**Affiliations:** 1 See Acknowledgments London United Kingdom; 2 Africa Eye Foundation Cameroon Yaoundé Cameroon

**Keywords:** ophthalmologic surgical procedures, access to health care, ophthalmology, patient-centered care, ethnography, health knowledge, attitudes, practice

*This is the authors’ response to peer-review reports for “Exploring the Reasons for Low Cataract Surgery Uptake Among Patients Detected in a Community Outreach Program in Cameroon: Focused Ethnographic Mixed Methods Study.”*

## Round 1 Review

Dear Editor and Reviewers,

We note with pleasure that your review comments were quite useful in helping us take a closer look and improve our work [1] further. We carefully observed and addressed all the comments as required and hope that the paper is in much better shape for the journal’s readership. Kindly find below our answers to all the editorial and reviewer comments. We will be happy to address any comments you may have further to the reviewed version of the manuscript.

### Reviewer P [2]

#### General Comments

Dear Reviewer P,

Thank you very much for the time you took to critically elaborate on the subject matter and for the compliments. We are grateful to you for indicating to us that this is an innovative paper. We also hold your views of extending our approach to other areas in which similar challenges are faced. Kindly find our answers to your comments below.

#### Minor Comments

1. It would be interesting if there are any other articles that mention this problem and can be added in the manuscript.

Response: We thank the reviewer for this suggestion. We took time to explore a journal database of community eye health [3] of articles dealing with barriers to the uptake of eye care services in similar settings published within the last 30 years. We found no article that fell within the last decade. However, we alluded to similar programs run in other countries in the second-to-last paragraph of the *Introduction* section (highlighted in yellow), and we carried out a comparison with similar studies in the *Discussion* section.

2. Moreover, the eye care delivery in Cameroon is presented only from the financial aspect. It would be interesting if the authors could add some other demographic or educational and cultural factors that affect the access to health care.

Response: We appreciate your concern. Apart from financial challenges, we also highlighted other factors that limit access to health care, which have now been substantiated. We also added a couple of lines, all of which have been highlighted in yellow.

### Reviewer Q [4]

#### General Comments

Dear Reviewer Q,

We are very grateful for the suggestions in improving our paper. We carefully considered and addressed all points as shown below.

#### Major Comments

1. It is better to choose keywords that are MeSH terms.

Response: We have modified the keywords to address this concern.

2. It is better to integrate all sections before *Methods* as an *Introduction* section.

Response: Done.

3. How did the researchers develop the interview guide?

Response: This has been clarified under *Data Collection Procedure* and highlighted in green.

4. The trustworthiness of the results and validity and reliability need to be discussed separately for each research method.

Response: We appreciate this suggestion. We did discuss the above under the subsection *Data Credibility*, which has been renamed as *Trustworthiness, Validity, and Reliability*. We have also made a few modifications.

5. More details should be added to the *Document Review* section.

Response: Done.

6. How many participants took part in the focus groups?

Response: All 29 subjects took part in the focus group discussions as highlighted in the table of participant characteristics. We have also rephrased the first sentence of the *FGDs* subsection to make this clearer.

7. The *Results* section needs to be expanded.

Response: Thank you for this. We were not able to expand the *Results* section owing to the editorial recommendation to reduce the length of the paper.

8. In the *Discussion* section, the summary of results does not need to be supported by the participant’s quotes.

Response: This has been removed.

9. The *Discussion* section needs to be revised to be more integrated.

Response: We have gone through the paper again and made corrections where necessary.

10. Strengths and limitations of the study can be reported at the end of the discussion section.

Response: Done.

11. Research implications can be reported before conclusions.

Response: Done.

### Reviewer BJ [5]

#### General Comments

Dear Reviewer BJ,

Thank you for taking the time to review our paper and for the recommendations. We considered all suggestions in improving the paper further. Kindly find below our responses to your comments.

#### Major Comments

1. The lengths of both the main text and the abstract are a bit long. We suggest the authors to further condense the paper or move some parts to Multimedia Appendices.

Response: We have removed some text from the *Abstract* and the body and maintained the word count in line with the guidelines [6].

2. Although 29 subjects were interviewed, only 9 of them were direct subjects. We are unsure if this is a sufficient number for such qualitative analysis.

Response: Thank you for raising this concern. In the context of this study, decisions regarding the uptake of cataract surgery to a greater extent are not made by blind patients with cataract themselves but rather by the breadwinner, if not the entire family, and often in consultation with other villagers who have been in similar situations, which sometimes may even extend to seeking advice from traditional healers or spiritualists about the success of the surgery. We wanted a sample that will represent the decision-making mechanism as highlighted under the *Ethnographic Rationale* subsection. This was discussed in a panel with colleagues, and it was determined that each subject category included in the sample played a key role in the uptake of cataract surgery.

There is evidence that data saturation in qualitative studies can be reached with a minimum sample of 13 [7-9]. The operated patients and blind patients with cataract together with their family members made up 15 subjects. According to Hennink and Kaiser [10], saturation can equally be reached with 9 subjects.

3. The influence of indirect subjects' opinions on the decision of the direct subjects was not particularly discussed.

Response: Thank you again for raising this. Following our explanation in point #2 above, it is a fact that direct patients to a lesser extent decide for themselves what they need. We have highlighted and underscored the fact that the decision-making mechanism in cataract surgery uptake is a social construct [11], with the family to a greater extent and the community to a lesser extent assuming major roles [12]. Kindly refer to the second-to-last paragraph of the *Conclusions* subsection (in pink).

4. Considering the potentially different weights of direct versus indirect subjects' opinions in the decision, whether the quotes were taken from direct subjects should be shown.

Response: Done.

5. We are no experts of traditional medicine, but is there anything to be noted about these therapies? (Maybe certain therapies were helpful from the patients' perspectives?) We are unsure if these should be taken into consideration when assessing the “Knowledge and awareness” and “reasons of refusal.”

Response: We have now added some text in the *Discussion* section to reflect this point (highlighted in pink).

6. The “poor outcome” of prior cataract surgeries was mentioned in the *Results* section. Can this be a possible reason for the “fear” of cataract surgery and the reason to choose traditional medicine instead?

Response: We have equally added a phrase under the *Perceived Reasons for Refusing Cataract Surgery* subsection in the *Discussion* section to reflect this.

#### Minor Comments

7. There are still some grammatical mistakes that should be checked and amended.

Response: We have read through and made some corrections.

8. Please make sure to provide the full spellings of all abbreviated words at first use (eg, “MICEI” and “FGDs”).

Response: Done

9. The table did not show the particular demographics of the direct subjects (which may help reveal other socioeconomic factors influencing the decision or limitation of the study).

Response: Done.

10. How is the surgery acceptance or backlog situation for community cataract screening programs conducted in nearby countries with a similar socioeconomic status? While this is not the focus of the study, if there are available data, it would be good to include some general information (this will help justify the study aim and support the overall results).

Response: Thank you for bringing this up. This has now been included in the second-to-last paragraph of the *Conclusions* subsection and highlighted in yellow.

## Round 2 Review

Dear Editor and Reviewers,

We thank you for pointing out the outstanding concerns which we have now carefully considered and addressed accordingly. We have now integrated our responses in the review comments for both rounds 1 and 2 as recommended by some of the reviewers.

### Reviewer Q

#### General Comments

Dear Reviewer Q,

We are thankful for the additional concerns. Kindly find our responses to your concerns below.

#### Major Comments

1. The author response letter only includes the authors’ responses without mentioning the reviewers' comments. For some comments, they just said “done” and I have no idea what the comments were and what they exactly did. So, a complete response letter needs to be uploaded.

Response: Our understanding in round 1 was that the reviewers had a copy of their comments. Additionally, we uploaded a copy of the response letter bearing the reviewer comments and our responses (as a supplementary file) and made it visible to the reviewers. We equally uploaded a version of the revised manuscript with track changes and made it visible to the reviewers as well. To address your concerns, we have included the responses for round 1 in this letter. We have also uploaded the revised manuscript with track changes.

2. The *Discussion* section needs to be integrated to show an integrated *Discussion* for the whole research. In the current format, it seems fragmented.

Response: We have now integrated the *Discussion* such that the former *Comparison With Prior Studies* is combined with the *Interpretation of Results* subsection, and we integrated the *Public Health Implications* subsection with the *Conclusions* subsection; we have made sure that our paper is in line with the journal guidelines with regard to the Discussion section [13].

3. Also, the subsections under the *Conclusions* section need to be moved to the end of the *Discussion* section or be integrated with other existing subheadings in this section.

Response: We have deleted the *Study Usefulness* subsection and integrated the *Recommendations* subsection with the *Conclusions* subsection as we think that the recommendations will better flow with the conclusion.

#### Reviewer BJ

Dear Reviewer BJ,

Thank you for taking the time to review our paper and for the recommendations. We considered all suggestions in improving the paper further. Kindly find below our responses to your comments.

1. The authors have addressed most of the comments. While the scientific content is acceptable after the revision, it is still recommended that the authors shortened the article to <6500-7000 words. No further suggestions are enclosed.

Response: We thank the reviewer for raising this concern. While we are not against cutting down the word count, we wish to reiterate that the word count is in line with the journal guidelines [6] that were updated 2 days ago. That notwithstanding, we have now down-worded the main body and abstract to 7329 words excluding the title, author information, multimedia appendices, references, and abbreviations. This is as opposed to a maximum of 10,000 words recommended [6].
